# Association Between Housing Insecurity, Psychological Distress, and Self-rated Health Among US Adults During the COVID-19 Pandemic

**DOI:** 10.1001/jamanetworkopen.2021.27772

**Published:** 2021-09-30

**Authors:** Sabriya L. Linton, Kathryn M. Leifheit, Emma E. McGinty, Colleen L. Barry, Craig Evan Pollack

**Affiliations:** 1 Department of Mental Health, Johns Hopkins University School of Public Health, Baltimore, Maryland; 2 Department of Health Policy and Management, University of California, Los Angeles Fielding School of Public Health, Los Angeles; 3 Department of Health Policy and Management, Johns Hopkins Bloomberg School of Public Health, Baltimore, Maryland; 4 Johns Hopkins School of Nursing, Baltimore, Maryland

## Abstract

This survey study examines association of housing insecurity with psychological distress and self-rated health among US adults during the COVID-19 pandemic.

## Introduction

Economic hardship due to the COVID-19 pandemic has exacerbated concerns about the threat of evictions and foreclosures.^1,2^ While prior research has documented an association between housing insecurity and health,^3^ the magnitude of this relationship has not been examined during the COVID-19 pandemic. Understanding the association between housing insecurity and health in a nationally representative sample during the pandemic is critical to inform efforts to support people harmed by the economic downturn.

## Methods

This survey study was deemed nonhuman participants research by the institutional review board at Johns Hopkins Bloomberg School of Public Health, and informed consent was waived. This study followed the American Association for Public Opinion Research (AAPOR) reporting guideline and the Survey Reporting Guideline (SURGE).

Data were from wave 3 of the Johns Hopkins COVID-19 Civic Life and Public Health Survey*,* conducted online November 11 to November 30, 2020, using the National Opinion Research Center’s probability-based AmeriSpeak panel. With a panel recruitment rate of 34%,^4^ AmeriSpeak is representative of the US civilian, noninstitutional adult population and covers 97% of households. A total of 1468 US adults participated in wave 1 (April 2020), which had a response rate of 70.4%. Among wave 1 respondents, 1337 responded to wave 2 (July 2020), and 1222 responded to wave 3 with completion rates of 91% and 92%, respectively.

Housing insecurity was defined as being currently behind on rent or mortgage or having no or low confidence in their ability to pay the next rent or mortgage payment vs having moderate or high confidence. Outcomes included psychological distress symptoms measured using Kessler 6 Psychological Distress Scale^5^ and dichotomized as moderate or severe vs lower psychological distress based on a score of 5 or more and self-rated health dichotomized as fair or poor vs good, very good, or excellent.

Covariates based on prior research^2,3,6^ included gender, race and ethnicity, age, household income in 2019, employment status, housing tenure, household structure, and urbanicity (eMethods in the Supplement). We separately estimated predicted probabilities of each outcome by status of housing insecurity, controlling for covariates. Analytic sample excluded 4 participants missing outcome data. Analyses were weighted to be nationally representative. Stata version 15 (StataCorp) was used for data analysis. Predicted probabilities were estimated from logistic regression models. Tests were 2-tailed and statistical significance was set at *P* < .05. Statistical analysis was performed between February to March 2021.

## Results

This survey study included 1218 participants (623 [51%] female; 560 [46%] aged 30 to 54 years; 549 [45%] aged 55 years or older; 161 [13%] self-identified as being Hispanic; 157 [13%] self-identified as being non-Hispanic Black; 841 [69%] self-identified as being non-Hispanic White). Overall, 128 participants (12%) reported housing insecurity in November 2020. Among participants experiencing housing insecurity, 42 participants (34%) reported being behind on housing payments, 55 participants (38%) reported having little to no confidence in their ability to make the next housing payment, and 31 participants (28%) reported both (Figure). Housing insecurity vs housing security was disproportionately higher among participants who rented their homes (78 [64%] vs 281 [27%]; *P* <.001); non-Hispanic Black race (44 [37%] vs 113 [8%]), were aged 30 to 54 years (88 [64%] vs 472 [38%]), earned less than $35 000 in 2019 (64 [52%] vs 268 [27%]), lived with children (58 [47%] vs 279 [29%]), or resided in metropolitan counties (116 [92%] vs 914 [84%]) (Table).

**Figure.**
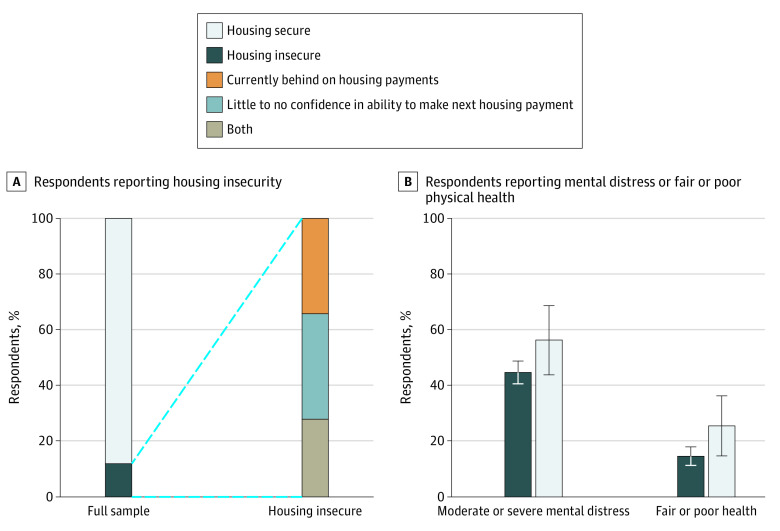
US Adult Survey Participants Reporting Housing Insecurity, Mental Distress, and Fair or Poor Physical Health in November 2020 The graphs display results for 1218 adults aged 18 years or older. Data from panel A are from wave 3 of the Johns Hopkins COVID-19 Civic Life and Public Health Survey, fielded November 11 to November 30, 2020. Adult survey participants were defined as having housing insecurity if they reported: (1) being currently behind on rent or mortgage or (2) having no or low confidence in their ability to pay the next rent or mortgage payment vs having moderate or high confidence. Analysis was unadjusted and weighted to be nationally representative. In panel B, psychological distress was measured using the Kessler 6 Psychological Distress Scale, with scores of 5 or higher indicating moderate or severe psychological distress. The error bars denote 95% CIs. Analyses were weighted to be nationally representative.

**Table.** Distribution of Economic and Sociodemographic Characteristics by Status of Housing Security Among a Nationally Representative Sample of US Adult Survey Participants in November 2020 (N = 1218)^a^

Characteristics	Housing, No. (%)		*P* value
	Secure (n=1090 [88])	Insecure (n=128 [12])	
Race and ethnicity			
Hispanic	136 (17)	25 (20)	<.001
Non-Hispanic			
Black	113 (8)	44 (37)	
White	791 (67)	50 (33)	
Other	50 (8)	9 (10)	
Gender			
Female	548 (51)	75 (58)	.28
Male	542 (49)	53 (42)	
Age group, y			
18-29	99 (19)	10 (14)	.001
30-54	472 (38)	88 (64)	
≥55	519 (43)	30 (21)	
Household income (2019), $			
<35 000	268 (27)	64 (52)	<.001
35 000-74 999	392 (33)	38 (26)	
≥75 000	430 (39)	26 (22)	
Currently unemployed	35 (4)	13 (10)	.007
Lost job or hours reduced due to COVID-19	212 (22)	44 (30)	.10
Household structure			
Single adult	192 (15)	17 (9)	.008
>1 Adult, no children	531 (47)	40 (37)	
>1 Adult, with children	279 (29)	58 (47)	
1 Adult with children	88 (9)	13 (8)	
Metropolitan area	914 (84)	116 (92)	.04
Renting their home %	281 (27)	78 (64)	<.001

^a^ Four individuals in the total analytic sample (N = 1222) were excluded because they were missing health outcome data (N = 2), either psychological distress or self-rated health (N = 2).

Forty-six percent (95% CI, 43%-50%) of participants reported severe to moderate psychological distress; 18% (95% CI, 15%-21%) reported fair to poor health. Compared with participants with housing security, participants experiencing housing insecurity reported higher distress (69 [57%] vs 467 [45%]) and lower self-rated health (33 [30%] vs 157 [16%]). After covariate adjustment, housing insecurity and lower self-rated health was statistically significant. The association between housing insecurity and higher distress was no longer statistically significant. Fifty-seven percent (95% CI, 44%-69%) of participants experiencing housing insecurity reported moderate or severe distress relative to 45% (95% CI, 41%-49%) of participants with housing security (*P* = .09), and 26% (95% CI, 15%-37%) of participants experiencing housing insecurity reported fair or poor health, relative to 15% (95% CI, 12%-18%) of participants with housing security (*P* = .03) (Figure).

## Discussion

In this nationally representative sample of US adults, housing insecurity was associated with higher psychological distress and lower self-rated health during the COVID-19 pandemic. The survey was conducted after the United States Centers for Disease Control and Prevention’s nationwide eviction moratorium, which may have attenuated these associations. Interventions that reduce housing insecurity during the COVID-19 pandemic will promote the health of the US population, and those interventions that consider equity in their implementation may mitigate entrenched health disparities resulting from structural racism and exacerbated by the pandemic. Results may be vulnerable to sampling biases, including the underrepresentation of adults experiencing homelessness. The cross-sectional design of this study precludes identifying causality; the survey did not assess prepandemic housing needs.

## Notes

Supplement.
eMethods.
