# Error in Figure

**DOI:** 10.1001/jamanetworkopen.2021.37473

**Published:** 2021-11-04

In the Research Letter titled “Association Between Housing Insecurity, Psychological Distress, and Self-rated Health Among US Adults During the COVID-19 Pandemic”^1^ published September 30, 2021, there was an error in the color scheme for Panel B in the Figure. The colors have been changed to correctly represent housing security and housing insecurity. This article has been corrected.^1^
