# Supplementary material for: Association Between Housing Insecurity, Psychological Distress, and Self-rated Health Among US Adults During the COVID-19 Pandemic
Source: JAMA Netw Open. 2021 Sep 30;4(9):e2127772. doi: 10.1001/jamanetworkopen.2021.27772 (PMC8485162; doi:10.1001/jamanetworkopen.2021.27772)
Supplement: Supplement. — eMethods. [file jamanetwopen-e2127772-s001.pdf]

Supplemental Online Content

Linton SL, Leifheit KM, McGinty EE, Barry CL, Pollack CE. Association between housing insecurity, psychological distress, and self-rated health among US adults during the COVID-19 pandemic. *JAMA Netw Open*. 2021;4(9):e2127772. doi:10.1001/jamanetworkopen.2021.27772

eMethods.

This supplemental material has been provided by the authors to give readers additional information about their work.

© 2021 Linton SL et al. *JAMA Network Open.*

eMethods. Survey Question Wording

Respondents were asked to answer questions 1 through 9 in wave 3 of the survey.

1. Is your house, apartment, or mobile home:
    a. Owned by you or someone in the household with a mortgage or loan?
    b. Owned by you or someone in this household free and clear, without a mortgage or loan?
    c. Rented?
    d. Occupied without payment of rent?

2. Which statement best describes your current employment status?
    a. Working full time for pay
    b. Working part time for pay
    c. Not working for pay – full or part time caregiver
    d. Not working for pay – on temporary layoff from a job
    e. Not working for pay – looking for work
    f. Not working for pay – retired
    g. Not working for pay – disabled
    h. Not working for pay – other

3. When the COVID-19 pandemic began in the U.S. in March 2020, which statement best described your employment status
    a. Working full time for pay
    b. Working part time for pay
    c. Not working for pay – full or part time caregiver
    d. Not working for pay – on temporary layoff from a job
    e. Not working for pay – looking for work
    f. Not working for pay – retired
    g. Not working for pay – disabled
    h. Not working for pay – other
    i. Don't know

4. Is your household currently caught up on [rent/mortgage] payments?
    a. Yes
    b. No
    c. Don't know

5. How confident are you that your household will be able to pay your next [rent/mortgage] payment on time?*
    a. No confidence
    b. Slight confidence
    c. Moderate confidence
    d. High confidence
    e. Payment is/will be deferred

© 2021 Linton SL et al. *JAMA Network Open.*

f. Don’t know

*Note: Participants reporting deferred payment were only counted as housing insecure if they also reported being behind on their rent (n=5), otherwise they were counted as housing secure (n=14).

6. In general, how would you rate your overall health?
    a. Excellent
    b. Very good
    c. Good
    d. Fair
    e. Poor
    f. Don’t know

Respondents were asked to answer questions 7 through 14 as baseline questions as part of their enrollment in NORC’s AmeriSpeak Panel.

For questions 7 through 13, respondents were given the following prompt: The next question is about the total income of YOUR HOUSEHOLD for [INSERT LAST YEAR EG: 2014 IF TODAY IS 2015]. Please include your own income PLUS the income of all members living in your household (including cohabiting partners and armed forces members living at home). Please count income BEFORE TAXES and from all sources (such as wages, salaries, tips, net income from a business, interest, dividends, child support, alimony, and Social Security, public assistance, pensions, or retirement benefits).

7. Was your total HOUSEHOLD income in [INSERT LAST YEAR]?
    a. Below $40,000
    b. $40,000 or more
    c. Don’t know

Respondents were prompted to answer question 8 only if they selected option “a” for question 7.

8. And was your total HOUSEHOLD income in [INSERT LAST YEAR]?
    a. Below $20,000
    b. $20,000 or more
    c. Don’t know

Respondents were prompted to answer question 9 only if they selected option “a” for question 8.

9. Which one of the following includes your total HOUSEHOLD income in [INSERT LAST YEAR] before taxes?
    a. Less than $5,000
    b. $5,000 to $9,999
    c. $10,000 to $14,999
    d. $15,000 to $19,999
    e. Don’t know

© 2021 Linton SL et al. *JAMA Network Open.*

Respondents were prompted to answer question 10 only if they selected option “b” for question 8.

10. Which one of the following includes your total HOUSEHOLD income in [INSERT LAST YEAR] before taxes?
    a. $20,000 to $24,999
    b. $25,000 to $29,999
    c. $30,000 to $34,999
    d. $35,000 to $39,999
    e. Don’t know

Respondents were prompted to answer question 11 only if they selected option “b” for question 7.

11. Was your total HOUSEHOLD income in [INSERT LAST YEAR]?
    a. Below $85,000
    b. $85,000 or more
    c. Don’t know

Respondents were prompted to answer question 12 only if they selected option “a” for question 11.

12. Which one of the following includes your total HOUSEHOLD income in [INSERT LAST YEAR] before taxes?
    a. $40,000 to $49,999
    b. $50,000 to $59,999
    c. $60,000 to $74,999
    d. $75,000 to $84,999
    e. Don’t know

Respondents were prompted to answer question 13 only if they selected option “b” for question 11.

13. Which one of the following includes your total HOUSEHOLD income in [INSERT LAST YEAR] before taxes?
    a. $85,000 to $99,999
    b. $100,000 to $124,999
    c. $125,000 to $149,999
    d. $150,000 to $174,999
    e. $175,000 to $199,999
    f. $200,000 or more
    g. Don’t know

14. Kessler Psychological Distress Scale (K6)

    a. During the past 30 days, about how often did you feel nervous?

© 2021 Linton SL et al. *JAMA Network Open.*

i. All of the time
ii. Most of the time
iii. Some of the time
iv. A little of the time
v. None of the time

b. During the past 30 days, about how often did you feel hopeless?
   i. All of the time
   ii. Most of the time
   iii. Some of the time
   iv. A little of the time
   v. None of the time

c. During the past 30 days, about how often did you feel restless or fidgety?
   i. All of the time
   ii. Most of the time
   iii. Some of the time
   iv. A little of the time
   v. None of the time

d. During the past 30 days, about how often did you feel so depressed that nothing could cheer you up?
   i. All of the time
   ii. Most of the time
   iii. Some of the time
   iv. A little of the time

e. During the past 30 days, about how often did you feel that everything was an effort?
   i. All of the time
   ii. Most of the time
   iii. Some of the time
   iv. A little of the time

f. During the past 30 days, about how often did you feel worthless?
   i. All of the time
   ii. Most of the time
   iii. Some of the time
   iv. A little of the time

© 2021 Linton SL et al. *JAMA Network Open.*
